# Emerging Roles for VEGF-D in Human Disease

**DOI:** 10.3390/biom8010001

**Published:** 2018-01-04

**Authors:** Steven A. Stacker, Marc G. Achen

**Affiliations:** 1 Tumour Angiogenesis and Microenvironment Program, Peter MacCallum Cancer Centre, 305 Grattan St., Melbourne, VIC 3000, Australia; 2 Sir Peter MacCallum Department of Oncology, University of Melbourne, Parkville, VIC 3010, Australia

**Keywords:** VEGF-D, lymphatic vessels, endothelium, metastasis, growth factor, receptor, signaling, angiogenesis, lymphangiogenesis

## Abstract

Blood vessels and lymphatic vessels are located in many tissues and organs throughout the body, and play important roles in a wide variety of prevalent diseases in humans. Vascular endothelial growth factor-D (VEGF-D) is a secreted protein that can promote the remodeling of blood vessels and lymphatics in development and disease. Recent fundamental and translational studies have provided insight into the molecular mechanisms by which VEGF-D exerts its effects in human disease. Hence this protein is now of interest as a therapeutic and/or diagnostic target, or as a potential therapeutic agent, in a diversity of indications in cardiovascular medicine, cancer and the devastating pulmonary condition lymphangioleiomyomatosis. This has led to clinical trial programs to assess the effect of targeting VEGF-D signaling pathways, or delivering VEGF-D, in angina, cancer and ocular indications. This review summarizes our understanding of VEGF-D signaling in human disease, which is largely based on animal disease models and clinicopathological studies, and provides information about the outcomes of recent clinical trials testing agonists or antagonists of VEGF-D signaling.

## 1. Introduction

Vascular endothelial growth factor-D (VEGF-D) is a secreted glycoprotein that can activate VEGF receptors on the endothelium, is a mitogen for endothelial cells and promotes the growth and remodeling of blood vessels and lymphatic vessels [1,2] (see Section 3 for further information). These vessels are located in many parts of the body and participate in a wide variety of prevalent human diseases. It is, therefore, not surprising that VEGF-D has been implicated in a multitude of disease processes from pulmonary indications to cancer. Furthermore, therapeutically targeting VEGF-D or its molecular signaling pathways in endothelial cells, or delivering VEGF-D to tissues, is considered therapeutically relevant to various clinical conditions. Therefore, there has been a major effort to develop reagents for manipulating VEGF-D signaling in diseases that are the subject of clinical trial programs in cancer, cardiovascular medicine and ocular indications (see Section 4 for further information). Importantly, insight into the function of this protein has led to improvements in the diagnosis of the devastating pulmonary disease lymphangioleiomyomatosis (LAM) [3,4] so research into the structure/function relationship, biochemistry and biology of VEGF-D in cell-based systems, animal models and clinicopathological studies has already led to improved clinical practice. Opportunities for further clinical impact are being developed through systems biology approaches to build a comprehensive understanding of the molecular signaling networks controlling the remodeling of blood vessels and lymphatics in disease [5,6], thereby enabling us to delineate how VEGF-D signaling is integrated with other signaling systems in the endothelial cells of blood vessels and lymphatics. These signaling systems are central to key biological processes in vascular biology such as cell proliferation, migration, tube formation and regression of excess vessels. Here we review the biology and clinical relevance of VEGF-D, with a particular focus on the potential impact of this insight on therapeutics and diagnostics for human disease.

## 2. Cloning, Gene Regulation and Biosynthesis of VEGF-D

**Figure 1:**
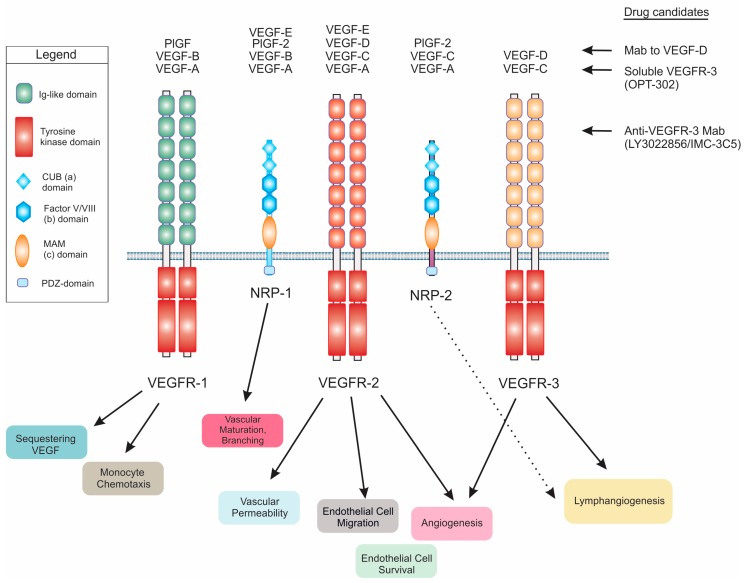
Schematic diagram illustrating the interaction of vascular endothelial growth factor-D (VEGF-D), and other VEGFs, with VEGF receptors (VEGFRs) and Neuropilin (NRP) co-receptors. Biological processes influenced by binding of ligands to the receptors are shown below, and selected drug candidates that can restrict VEGF-D signaling are listed to the right with “Mab” denoting monoclonal antibody. Note that soluble VEGFR-3 and anti-VEGFR-3 Mab also modulate VEGF-C signaling. “PlGF” denotes placenta growth factor, Ig denotes immunoglobulin, CUB denotes complement-binding, PDZ denotes a domain found in post synaptic density protein (PSD95)/Drosophila disc large tumor suppressor (Dlg1)/zonula occludens-1 protein (zo-1) and MAM denotes domain with homology to meprin A5 and mu-phosphate.

VEGF-D was first identified as a c-fos-induced growth factor (FIGF), the expression of which was induced by the transcription factor c-fos, via mRNA differential screening of fibroblasts differing in the expression of c-fos [7]. Subsequently, VEGF-D was shown to be a ligand for VEGF receptor (VEGFR)-2 and VEGFR-3 (Figure 1), cell surface receptor tyrosine kinases expressed on blood vessels and lymphatic vessels [2]. VEGF-D activates both VEGFR-2 and VEGFR-3, is a mitogen for blood vascular and lymphatic endothelial cells and is closely related to VEGF-C (another member of VEGF family of ligands) from a structural perspective [2,8]. VEGF-D, as with other members of the VEGF family, falls within a structural superfamily of growth factors containing a cystine knot motif, which involves a highly distinctive clustered arrangement of three intrasubunit cystine bridges [9]. Most members of the VEGF family exist as covalent-linked homodimers, however, VEGF-D can also exist as a non-covalent homodimer as well as a monomer [10]. The crystal structure of a covalent VEGF-D homodimer, similar to other VEGF family members, involves monomers with an antiparallel 4-stranded β-sheet, three connecting loops and an N-terminal α-helix that folds on top of the second monomer [11]. This antiparallel homodimer is stabilized by two intersubunit disulfide bridges.

The gene encoding VEGF-D (known as the *FIGF* gene) is expressed in a range of tissues during development, and in adult tissues, with prominent expression in lung and skin [2,10]. However, the molecular mechanisms that regulate expression of the *FIGF* gene are not fully understood. The c-FOS transcription factor and cell–cell contact mediated by cadherin-11 have each been shown to promote expression of VEGF-D in fibroblasts [7,12]. Hence, molecular signaling pathways that operate via c-FOS can induce expression of VEGF-D—an example is the capacity of interleukin 7 to promote VEGF-D production in a range of cancer cells via a c-FOS-dependent pathway [13,14]. Conversely, expression of the *FIGF* gene can be downregulated by transforming growth factor-β1 in fibroblasts via the Jun NH_2_-terminal kinase signaling pathway [15]. Moreover, the interaction of the orphan receptors hepatocyte nuclear factor 4α (HNF-4α) and chicken ovalbumin upstream promoter transcription factor (COUP-TF)-1/COUP-TF-2 with a proximal element of the *FIGF* gene are indispensable for transcription from this gene [16]. Expression of VEGF-D can also be modulated at the mRNA level as indicated by the finding that β-catenin can inversely regulate the stability of VEGF-D mRNA [17].

The biosynthesis of VEGF-D can involve extracellular proteolytic processing [10]. VEGF-D is initially secreted from the cell as a full-length form consisting of a central domain, containing receptor-binding sites, flanked by N- and C-terminal propeptides [2,10]. The propeptides can be cleaved by members of the proprotein convertase family of proteases [18], or plasmin [19,20], to generate various forms of VEGF-D, including a mature form lacking both propeptides [10]. This proteolytic processing, which occurs in mouse [21] and man [10], regulates VEGF-D signaling given that the differently processed forms have distinct receptor-binding and activation properties. For example, the mature form of VEGF-D can bind and activate both VEGFR-2 and VEGFR-3 whereas full-length unprocessed VEGF-D has very poor affinity for VEGFR-2 and binds VEGFR-3 but with less favourable affinity than mature VEGF-D [18]. Thus, the proteolytic processing of VEGF-D regulates its bioactivities.

## 3. Receptor Signaling and Biological Function

### 3.1. Receptor Signaling

The mature form of human VEGF-D can promote both angiogenesis (the sprouting and growth of new blood vessels) and lymphangiogenesis (the growth of lymphatic vessels) via activation of VEGFR-2 and VEGFR-3 on endothelial cells (Figure 1) [2,22]. VEGFR-2 has been considered the key receptor for signaling for angiogenesis whereas VEGFR-3 is critical for lymphangiogenesis (detailed reviews of these receptors, associated co-receptors and mechanisms of activation are presented elsewhere [23,24]). However, VEGFR-3 can contribute to angiogenic signaling by promoting conversion of endothelial cells from a tip cell to a stalk cell phenotype at the fusion points of blood vessel sprouts [25]. Moreover, blocking VEGFR-3 can suppress sprouting of blood vessels [26]. Importantly, it has been shown that VEGFR-2 signaling can play a role in modulating lymphatics as it is able to induce lymphatic vessel enlargement but not sprouting [27]. In addition to signaling via VEGFR-2 or VEGFR-3, VEGF-D can also signal via VEGFR-2/VEGFR-3 heterodimers [28]. Signaling via these heterodimers is thought to promote VEGF-D-driven dilation of collecting lymphatic vessels in cancer by down-regulating expression of the enzyme 15-hydroxyprostaglandin dehydrogenase, which degrades prostaglandins, in lymphatic endothelial cells [29,30]. In summary, signaling from either VEGFR-2 or VEGFR-3 can influence remodeling of both blood vessels and lymphatics [23,24].

### 3.2. Biological Function

**Figure 2:**
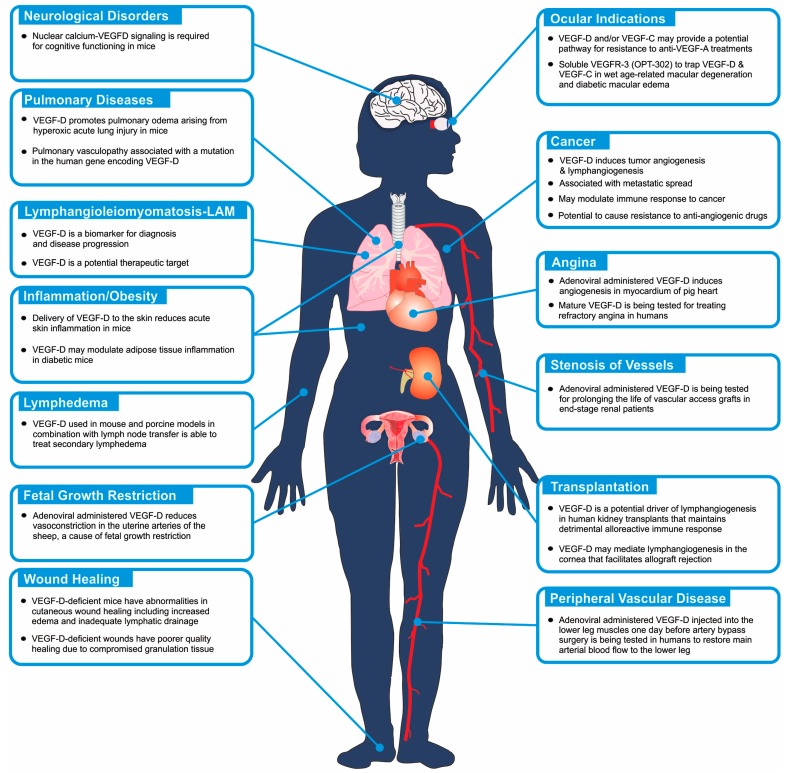
Schematic diagram illustrating areas of the human body that may have VEGF-D-dependent biological and pathological processes. The diagram highlights those biological processes that have been demonstrated to be involved in human pathology (see text for references), and indicates where VEGF-D signaling has been demonstrated to be relevant based on animal models, or has been used as a target for therapy or as a biomarker of a disease or the progression of a disease.

The role of VEGF-D in embryonic development has been explored in mouse, *Xenopus laevis* (frog) and *Danio rerio* (zebrafish) using gene knockout and gene knockdown approaches. In the mouse, inactivation of the gene encoding Vegfd revealed that this protein is dispensable for development of the lymphatic system [31,32,33] but plays subtle roles in modulating lymphatics in skin [34] and enhancing the density of lymphatics adjacent to bronchioles in the lung [31]. Further, VEGF-D could regulate blood vascular development by modulating the activity of the SOX18 transcription factor [35]. VEGF-D exhibited a subtle modifier role in embryonic lymphangiogenesis in *Xenopus*, contributing to migration of lymphatic endothelial cells [36]. In zebrafish, VEGF-D modulates both angiogenesis and lymphangiogenesis during embryonic development [37], and can compensate for the loss of VEGF-C for the sprouting of facial lymphatics [38]. Overall, VEGF-D appears to play a subtler role in regulating lymphatic vascular development than VEGF-C (which is absolutely required for lymphatic development [39]), and can participate in regulating blood vascular development particularly in association with SOX18. VEGF-D can also play important roles in the biology underlying a variety of disease settings (Figure 2), which are described in the following sections.

## 4. Disease Involvement: Humans and Model Systems

### 4.1. Lymphangioleiomyomatosis

Lymphangioleiomyomatosis is a rare pulmonary disease that typically arises in women during their child-bearing years [40]. This potentially lethal condition is a low-grade metastasizing neoplasm [41], which involves proliferation of abnormal muscle-like cells (LAM cells), harboring inactivating mutations in the tumor suppressor genes encoding tuberous sclerosis proteins 1 or 2 (*TSC1* or *TSC2* genes) [42], along the axial lymphatics and in the lungs. This leads to extensive pulmonary lymphangiogenesis, formation of diffuse cysts and destruction of lung tissue that can result in leakage of chyle [40]. LAM cells produce and secrete VEGF-D [43], which may play a role in disease progression by promoting association of LAM cells with lymphatic endothelial cells thus facilitating spread of LAM cells to the lung [44,45,46]. The VEGF-D produced by LAM cells may also promote pathological lymphangiogenesis associated with the condition. Importantly, serum levels of VEGF-D can be a surrogate marker for LAM severity [43] as well as a measure of lymphatic involvement [47]. VEGF-D serum levels can also be used to distinguish LAM from other pulmonary conditions [3]. Hence, a serum test for VEGF-D is being used as a diagnostic for LAM and for monitoring the efficacy of potential therapeutics for treating this condition [4,48,49]. The drug Sirolimus (also known as rapamycin; trade name is Rapamune, Pfizer Inc., New York City, NY, USA), which is an inhibitor of the kinase mTOR (mTOR is also known as a mammalian target of rapamycin and is a component of the mTORC1 signaling complex that can be regulated by TSC1 and TSC2 [50]), is now used to treat LAM and can stabilize lung function over many years of therapy while producing a sustained reduction of VEGF-D levels [51,52]. In addition to diagnostics and disease monitoring, the potential role of VEGF-D in LAM progression indicates that this protein, or other components of its signaling pathway, might be a relevant therapeutic target in this disease. 

### 4.2. Pulmonary Diseases

Recent findings have implicated VEGF-D in the pathogenesis of other pulmonary diseases. For example, a form of pulmonary vasculopathy, involving dilated or distended pulmonary arteries and veins, has been associated with a mutation of the gene encoding VEGF-D in humans [53]. The mutation resulted in increased dimerization of VEGF-D (VEGF-D dimers are considered more bioactive than VEGF-D monomers [10]), elevated VEGFR-2 signaling and aberrant angiogenesis. Moreover, VEGF-D was shown to promote pulmonary edema in a mouse model of hyperoxic acute lung injury (HALI), a form of injury that can occur when patients are ventilated with high concentrations of oxygen [54]. The detrimental effect of VEGF-D in the HALI model appeared to be due to enhanced fluid leakage from blood vessels. The proposed roles of VEGF-D in LAM, pulmonary vasculopathy and pulmonary edema demonstrate the capacity of this growth factor to influence disease processes by modulating pulmonary blood vessels or lymphatics.

### 4.3. Cardiovascular Diseases

**Figure 3:**
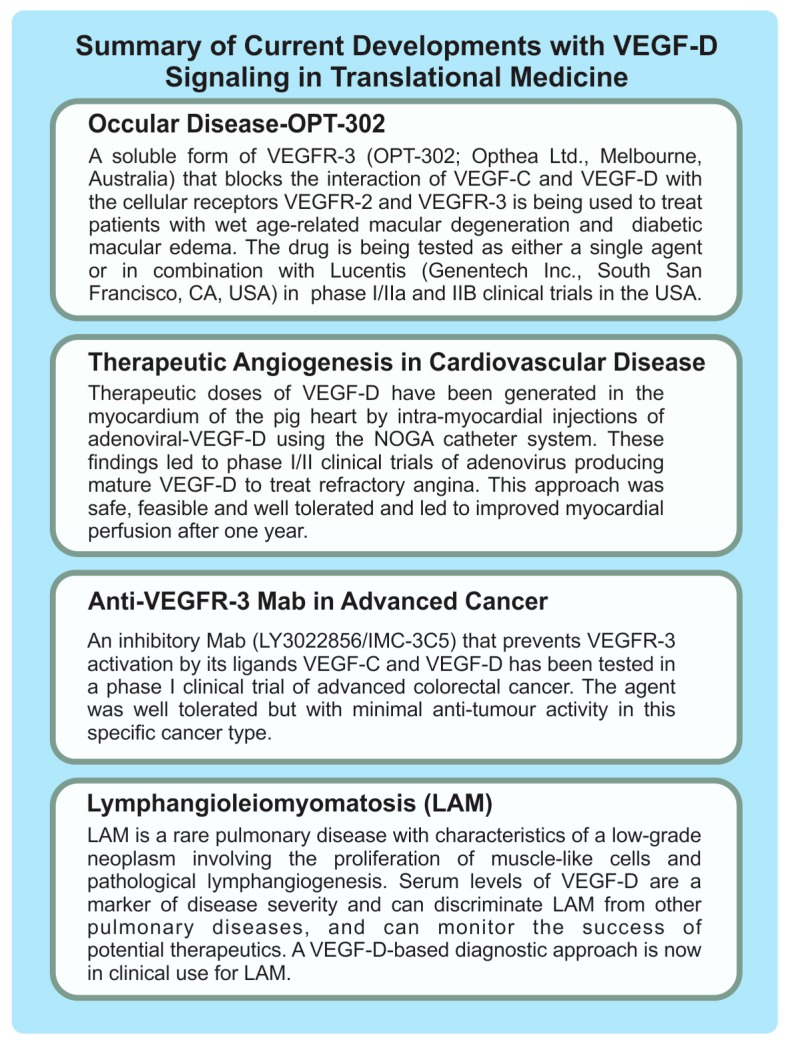
Summary of current and potential therapeutic and diagnostic applications of the VEGF-D signaling pathway in human disease. The diagram includes information on recent clinical trials and the use of diagnostic tests. Further information and references are included in the text of this article.

The therapeutic benefits of various forms of human VEGF-D have been assessed in animal models of prevalent cardiovascular diseases, via viral-based gene delivery approaches, which led to a range of clinical trials in cardiovascular medicine (Figure 3). For example, an adenovirus expressing a mature form of VEGF-D was injected into the myocardium of pig heart using the NOGA catheter system (Biosense-Webster, Johnson & Johnson, Irvine, CA, USA) resulting in transmural angiogenesis that was most pronounced in the epicardium [55]. This finding, suggesting that mature VEGF-D could be used to drive therapeutic angiogenesis in the heart, led to phase I/IIa clinical trials of an adenovirus producing mature VEGF-D for treating refractory angina. This study showed that intramyocardial delivery of mature VEGF-D via adenoviral gene transfer in refractory angina patients was safe, feasible and well tolerated [56]. Importantly, this therapeutic approach led to increased myocardial perfusion after one year. The study also identified that plasma lipoprotein(a) may be a potential biomarker to identify patients who experience the greatest benefit with this therapy. These encouraging results may justify phase IIb/III clinical trials to confirm the safety and efficacy of this gene therapy in refractory angina patients.

Viral gene transfer of human VEGF-D has been studied in animal models of a variety of other cardiovascular indications including vascular restenosis [57], peripheral vascular disease, and reduced uterine artery blood flow leading to fetal growth restriction [58], with promising results. This approach has also been shown to promote angiogenesis in skeletal muscle in rabbits [59,60], which could be relevant to clinical situations in which occluded arteries or ischemic tissues cannot be treated by angioplasty, stenting or by-pass-surgery. Notably, intradermal injection of mature human VEGF-D protein induced angiogenesis in the skin of sheep [61], indicating the potential of an alternative VEGF-D delivery approach to viral gene transfer. A range of clinical trial programs have begun to explore the benefit of VEGF-D in vascular restenosis, peripheral vascular disease and fetal growth restriction (Figure 3) although outcomes from early-stage trials are yet to be reported as far as we are aware.

### 4.4. Ocular Indications

Wet age-related macular degeneration (wet AMD; also known as neovascular or exudative AMD) is a common cause of vision impairment and blindness in the developed world, particularly in older people. It involves the formation of abnormal, tortuous blood vessels under the macula, which can bleed and leak fluid leading to macular damage that, if untreated, can cause severe loss of central vision. Proliferation of blood vascular endothelial cells and vessel leakage leading to edema are key features of this problematic condition [62]. Currently, the most effective treatments for wet AMD are anti-angiogenic drugs that principally target VEGF-A, including Lucentis (also known as ranibizumab; Genentech, Inc., South San Francisco, CA, USA), Avastin (bevacizumab; Genentech, Inc.) and Eylea (aflibercept; Regeneron Pharmaceuticals, Inc., Tarrytown, NY, USA), that are delivered by injection into the eye. These drugs likely restrict both the VEGF-A-driven proliferation of vascular endothelial cells and the enhanced vascular permeability promoted by VEGF-A. However, not all patients respond to these drugs, some patients exhibit responses that are considered sub-optimal and others initially respond well but eventually experience acquired drug resistance. Hence there is considerable interest in drugs that might be used in combination with Lucentis, Avastin or Eylea to improve clinical outcomes [63]. Given that VEGF-C and VEGF-D can activate signaling for proliferation of vascular endothelial cells and angiogenesis [2,64], and have been reported to promote edema in certain animal models [54,65], there has been interest in targeting these growth factors in wet AMD as a strategy to further enhance the restriction of pathological neovascularization and vascular leakage achieved with currently used drugs. To this end, a soluble form of VEGFR-3 (designated OPT-302; Opthea Ltd., Melbourne, Australia), designed to block the biological effects of both VEGF-C and VEGF-D (Figure 1), is being tested in phase I/IIa clinical trials of wet AMD in the USA. OPT-302 is being delivered by intraocular injection and tested as a single agent or in combination with Lucentis (Figure 3). Thus far, the trials have demonstrated the safety and tolerability of OPT-302 as monotherapy and in combination with Lucentis, and suggested that combined administration of OPT-302 and Lucentis may lead to improved clinical outcomes over Lucentis alone [66].

There is a range of other ocular indications for which drugs targeting VEGF-A have been approved by regulatory authorities. For example, Lucentis has been approved for treating macular edema following retinal vein occlusion, diabetic macular edema, diabetic retinopathy in patients with diabetic macular edema and myopic choroidal neovascularization. The degree to which VEGF-D is involved in resistance mechanisms to anti-VEGF-A drugs in these indications is unknown but warrants investigation.

### 4.5. Cancer

In experimental settings, VEGF-D promoted angiogenesis and lymphangiogenesis within and at the periphery of solid tumors, as well as enhancing solid tumor growth and metastatic spread to lymph nodes and distant organs, in a range of mouse cancer models including transgenic, gene ablation and xenograft models [1,32,67,68,69,70,71]. In addition to promoting the growth of small lymphatics adjacent to primary tumors, thus facilitating entry of tumor cells into the lymphatic vasculature, VEGF-D also promoted metastasis via dilation of collecting lymphatic vessels, which likely facilitates transport of tumor cells through the lymphatic vasculature [29]. These findings indicate the multi-faceted effects of VEGF-D on tumor biology. The capacity of VEGF-D to drive dilation of collecting lymphatics is dependent on prostaglandins; this dilation, and the associated increase in metastatic spread, could be blocked by Etodolac, a non-steroidal anti-inflammatory drug (NSAID) that ablates prostaglandin production [29]. Hence, NSAIDs could exhibit an anti-cancer effect by restricting metastasis via lymphatics, potentially explaining the anti-cancer effects of the NSAID aspirin observed in randomized clinical trials and other studies [72,73,74,75,76]. VEGF-D, as well as VEGF-C, were recently shown to play a potential role in lymphangiogenesis in ovarian cancer as the secreted protein acidic and rich in cysteine (SPARC), a calcium-binding glycoprotein, appears to function as a tumor suppressor in this disease by inhibiting angiogenesis and lymphangiogenesis by reducing expression of both VEGF-C and VEGF-D [77]. While the source of VEGF-D in the animal models referred to above has typically been the tumor cell, VEGF-D has been associated with both tumor cells and infiltrating immune cells in human tumors [78].

The relevance of VEGF-D to human cancer is supported by clinicopathological data indicating that its expression can correlate with metastatic spread and poor patient outcomes [79,80]. For example, VEGF-D was reported as an independent predictor of poor outcome in epithelial ovarian carcinoma [81] and a prognostic marker for disease-free and overall survival in colorectal carcinoma [82]. Further, VEGF-D and VEGFR-3 were reported to be independent prognostic markers aiding identification of patients with poor prognosis after curative resection of gastric adenocarcinomas [83]. Interestingly, a clinical study of Avastin (an anti-VEGF-A antibody) in metastatic colorectal cancer indicated that low VEGF-D expression was associated with greater benefit from Avastin in terms of progression-free and overall survival [84]. High expression of VEGF-D was predictive of resistance to Avastin, particularly in terms of progression-free survival. The predictive value of VEGF-D appeared to depend on the chemotherapy used in combination with Avastin. These findings provide further impetus for targeting VEGF-D signaling in human cancer.

Recent studies have demonstrated that lymphatic vessels and lymphatic remodeling can influence the immune response to cancer. For example, VEGF-C was shown to promote immune tolerance in a melanoma model in mice and to enhance cross-presentation of tumor antigen by lymphatic vessels in lymph nodes [85]. These findings indicate the possibility of targeting lymphangiogenic signaling by growth factors such as VEGF-C or VEGF-D to counter immune tolerance to cancer. This is currently an area of active fundamental research in cancer biology with potential consequences for immunotherapeutic strategies [86,87,88].

A range of reagents to target VEGF-D or components of its signaling pathways has been developed, including neutralizing monoclonal antibodies (Mabs) to VEGF-D [8,89,90] or to VEGFR-3 [91,92,93], and soluble forms of VEGFR-3 [94,95,96,97,98] (Figure 1), that restricted solid tumor growth and/or metastatic spread in animal models of cancer. It should be noted that antibodies to VEGFR-3 can restrict activation of this receptor by either VEGF-C or VEGF-D, and that soluble forms of VEGFR-3 can sequester both of these growth factors. A phase I clinical trial of a VEGFR-3 Mab, designated LY3022856/IMC-3C5, in patients with advanced and refractory solid tumors and advanced colorectal cancer recently demonstrated that this agent was well tolerated but with minimal anti-tumor activity in colorectal cancer [99]. This lack of efficacy highlights the importance of developing biomarker-based strategies to identify patients who would be more likely to respond to clinical agents targeting VEGF-C or VEGF-D signaling pathways. Given the importance of the proteolytic processing of VEGF-D for its capacity to promote the growth and spread of tumors [100,101], the enzymes that process VEGF-D [19] such as proprotein convertases [18] are potential targets for anti-cancer therapeutics designed to restrict tumor angiogenesis and lymphangiogenesis. However, targeting theses enzymes may have a multiplicity of effects in cancer, and a difficult-to-predict range of side-effects, because they can influence a range of signaling pathways, i.e., their action is not restricted to modulating only VEGF-D signaling.

### 4.6. Inflammation and Obesity

The effects of VEGF-D have been explored in various animal models of inflammation. For example, delivery of VEGF-D via transgenesis reduced acute skin inflammation in a mouse model that was associated with a significant reduction of edema in the dermis [102]. Findings such as these have suggested that stimulating lymphangiogenesis, using VEGF-C or VEGF-D, might be a new approach to combat chronic inflammatory diseases of the skin and other organs [103]. A study of chronic airway inflammation in mice, involving lymphatic vessel hyperplasia, showed that impairment of lymphangiogenesis could lead to bronchial lymphedema and exaggerated obstruction of the airways [104]. Perhaps correction of defective lymphangiogenesis may be beneficial in asthma and other inflammatory conditions.

Studies in a diabetic mouse model showed that blockade of VEGF-C and VEGF-D with a soluble form of VEGFR-3 can modulate adipose tissue inflammation, which was associated with reduced hepatic lipid accumulation and improved insulin sensitivity indicating an unanticipated function of lymphangiogenic factors in mediating metabolic syndrome-associated adipose tissue inflammation [105]. Targeting this signaling pathway may therefore be a therapeutic approach for preventing obesity-associated insulin resistance.

### 4.7. Lymphedema

Lymphedema is an edema usually caused by lymphatic abnormalities that compromise uptake of fluid from the interstitium of tissues. Primary lymphedema includes conditions with a genetic origin (often involving abnormal development of the lymphatic vasculature) whereas secondary lymphedema, which is more common, can arise from damage to lymphatic vessels caused by surgery (e.g., removal of lymph nodes), infection, trauma or radiation therapy [106]. The condition is characterized by debilitating swelling of tissue, typically in the limbs, which can be highly problematic for patients both physically and psychologically. There are no effective treatments for this condition so a variety of molecular-based strategies to enhance lymphatic drainage in this disease setting are being explored. The potential relevance of VEGF-C or VEGF-D signaling to lymphedema is illustrated by the recent finding of a single nucleotide polymorphism in the gene encoding VEGFR-3 that is associated with the clinical development of lymphedema caused by lymphatic filariasis [107].

A promising approach being tested in secondary lymphedema is the combination of autologous lymph node transfer with viral-driven growth factor expression to promote lymphatic regeneration or repair and thereby facilitate survival of the transplanted lymph node. In a mouse model, treatment of lymph node-excised animals with VEGF-D, delivered by adenovirus, promoted the growth of small lymphatics, which differentiated into functional collecting lymphatics, or remodeling of pre-existing collecting lymphatics, that were associated with an improved outcome of lymph node transplantation [108]. Since then, this approach has been assessed in a porcine model of secondary lymphedema, demonstrating that lymphatic drainage was significantly improved in animals treated with VEGF-C or VEGF-D. These findings indicate that this approach should be tested in clinical trials for treatment of secondary lymphedema [109].

### 4.8. Other Clinical Settings

#### 4.8.1. Wound Healing

Genetically modified mice deficient for Vegf-d have been reported to exhibit abnormalities in cutaneous wound healing compared to wild-type controls. In particular, the wound epithelium of Vegf-d-deficient mice was more edematous and thicker, likely reflecting inadequate lymphatic drainage [110]. Further, myofibroblasts were more abundant in Vegf-d-deficient wounds leading to faster wound closure, but resorption of granulation tissue was compromised, which is consistent with a poorer-quality of healing. These findings raise the possibility that VEGF-D could therapeutically enhance the quality of healing of difficult-to-treat cutaneous wounds, but this needs to be tested in more clinically relevant animal models.

#### 4.8.2. Transplantation

It has been proposed that lymphangiogenesis facilitates rejection of transplanted tissue in various clinical settings. For example, it was shown that a soluble form of VEGFR-3, capable of sequestering VEGF-C and VEGF-D, suppressed both lymphangiogenesis in the cornea and allograft rejection suggesting this may be a viable therapeutic approach to enhance corneal allograft survival [111]. In human kidney transplants, lymphangiogenesis was observed to be associated with immunologically active lymphocytic infiltrates, leading to speculation that lymphangiogenesis is involved in maintenance of a detrimental alloreactive immune response [112]. These, and similar findings, have led to the proposal that lymphatic neoangiogenesis may be a driving force of chronic rejection of renal transplants [113]. It is also possible that VEGF-C and VEGF-D may act directly on immune cells to modulate their action in transplantation but further research is required to explore this possibility.

#### 4.8.3. Neurological Disorders

In addition to acting on the endothelium, VEGF-D has been shown to play a role in the biology of neurons. More specifically, VEGF-D can control the length and complexity of dendrites in cultured hippocampal neurons and in adult mouse hippocampus, and, importantly, nuclear calcium-VEGF-D signaling was required for the effect of neuronal activity on maintaining dendritic arbors in adult hippocampus and for cognitive functioning [114,115]. More recently it was demonstrated that nuclear calcium, acting via the gene encoding VEGF-D, is required for hippocampus-dependent fear memory consolidation and extinction [116]. These findings suggest that VEGF-D could be used therapeutically to stabilize dendritic structures and network connectivity, thereby preventing cognitive decline, and could be beneficial in psychiatric disorders, neurodegenerative and aging-related conditions involving loss of neuronal structures [116].

## 5. Concluding Comments

Fundamental research into the biochemistry, molecular signaling and biological functions of VEGF-D has been central to identifying the potential roles of this angiogenic and lymphangiogenic growth factor in a range of clinical settings. The production of agents for targeting or delivering this protein, such as monoclonal antibodies and recombinant adenoviruses, has led to translational research and ongoing clinical trial programs assessing the benefits of targeting VEGF-D signaling pathways, or delivering VEGF-D, in cancer, cardiovascular medicine and ocular indications. Importantly, the recognition that monitoring serum levels of VEGF-D can be useful for diagnosing LAM has led to a new diagnostic approach for this disease that is proving useful in combination with other strategies. Hence, research into the biochemistry and biology of VEGF-D has already changed clinical practice and, with multiple clinical trial programs underway, it is reasonable to propose there may be more clinical impact to come.
